# D-dimer trends predict COVID-19 patient’s prognosis: A retrospective chart review study

**DOI:** 10.1515/med-2023-0816

**Published:** 2023-10-16

**Authors:** Raeed Kabir, Iyana Malik, Reena Chen, Jebun Nahar, Eagle Chen, Sheikh M. Hoq, Azad Kabir

**Affiliations:** Department of Research and Innovation, Doctor Ai, LLC, MS 39530, USA; Jackson Hospital, Montgomery, Alabama, USA; Augusta Health, Fishersville, Virginia, USA; Department of Research and Innovation, Doctor Ai, LLC, 1120 Beach Blvd, Biloxi, MS 39530, USA

**Keywords:** COVID-19, SARS-CoV-2, anticoagulation, enoxaparin, bleeding risk, prognosis, oxygen, vaccination, D-dimer, non-critically ill

## Abstract

This is a retrospective study of patients admitted to Jackson Hospital, Montgomery, Alabama, with a diagnosis of COVID-19 from January 1, 2021, to February 15, 2022. The independent variables used in the models were patient sex, age, race, BMI category, daily D-dimer categories, categories of anticoagulation doses, bleeding episodes, and vaccination status. The three different categories of anticoagulation doses were considered for the purpose of the study which were Enoxaparin 40 mg daily vs Enoxaparin 80 mg daily vs Enoxaparin 1 mg/kg or equivalent daily. The study reviewed a total of 100 hospitalized patients. Intermediate-dose anticoagulation was found to be the optimal dose as only 14% patients died compared to a 36 and 50% death rate among those treated with low-dose and high-dose anticoagulation, respectively. The multivariate linear regression model predicting patient oxygen requirements revealed D-dimer and bleeding status to be statistically significant predictors with a *p* value of <0.01. For the patients who had a D-dimer value ≥2 µg/mL, the oxygenation requirement was predicted to be 31 L higher than those with a D-dimer <2 µg/mL (99% CI; *p* < 0.01). When mean D-dimer and corresponding oxygen requirements were calculated per hospitalization days category, the D-dimer levels and oxygen requirements were noted to follow the same trends indicating that both values tended to increase and decrease simultaneously. The study concludes daily D-dimer trends can predict COVID-19 patient survival or daily oxygen requirements indicating that D-dimer can be the miracle molecule for COVID-19 prognosis.

## 1 Introduction

Three large randomized clinical trials named the ATTACC, ACTIV-4a, and REMAP-CAP [1] were terminated early because the study conclusively found that noncritically ill patients with COVID-19 on therapeutic-dose anticoagulation with heparin had an increased probability of survival as compared with usual-care thromboprophylaxis. The study also showed that major bleeding occurred in 1.9% of the patients receiving therapeutic-dose anticoagulation, and progression to intubation or death occurred in 10.9% (129 out of 1,181 patients) of the therapeutic-dose anticoagulation group. The study projected that for every 1,000 hospitalized patients with moderate disease, an initial strategy of therapeutic-dose anticoagulation, as compared with usual-care thromboprophylaxis (5,000 units SQ q12), would be anticipated to result in the survival of 40 additional patients until hospital discharge without organ support at the expense of 7 additional major bleeding events [1].

The REMAP-CAP, ACTIV-4a, and ATTACC Investigators also reported that 40.9% (217 patients out of 531) of patients had major thrombotic events or deaths among all of the patients who were treated with therapeutic dose anticoagulation in the intensive care unit. Among these patients, 3.8% (20 patients out of 529) had major bleeding. The phenomenon of increased risks for bleeding, thrombotic events, or death among the patients admitted to critical care units caused nationwide intensivists to be reluctant to include therapeutic anticoagulation in their treatment choice for COVID-19 patients [2].

A prior autopsy study showed significantly increased endothelial injury (endotheliitis), widespread thrombosis with microangiopathy, and alveolar-capillary microthrombi in the lungs of the patients who died from COVID-19 compared to the lungs of individuals who died of influenza or other causes [3]. Another autopsy study of 21 individuals with COVID-19 showed prominent pulmonary emboli (PE) in 19% of cases and microthrombi in the alveolar capillaries in 45% of cases. Interestingly, the primary cause of death in each of the cases was found to be caused by respiratory failure due to exudative diffuse alveolar damage and massive capillary congestion accompanied by microthrombi [4].

It is possible that an overwhelming burden of microthrombi leads to acute respiratory distress syndrome or death in patients with COVID-19 since anticoagulation does not dissolve the thrombi but rather helps prevent the thrombi from growing larger. In such cases, it is expected to have increasing trends of D-dimer, along with high oxygen requirements, among patients who are critically ill with COVID-19.

This study tested the hypothesis that increasing trends of D-dimer are associated with an increase in oxygen requirements among patients with COVID-19. If this hypothesis is found to be true, then D-dimer can be used to predict COVID-19 patient survival. In such a case, a low or moderate dose anticoagulant should be started at the onset of the COVID-19 diagnosis to reduce the risk of bleeding related to therapeutic anticoagulation and the anticoagulation dose should be adjusted based on D-dimer trends.

The current retrospective study was designed to test the above hypothesis as well as if administration of intermediate dose anticoagulation could be related to increased survivability compared to the patients who received low- or high-dose anticoagulation.

## 2 Methods

### 2.1 Study population

This study retrospectively evaluated total of 100 COVID-19 admission at Jackson Hospital, Alabama, USA, from January 1, 2021, to February 15, 2022, for patients who had D-dimer levels in the medical chart. All patient charts were systematically reviewed by two investigators where findings were matched and evaluated for discrepancies that were resolved. The following variables were collected: sex, age, race, BMI, daily D-dimer values, associated oxygenation requirements, anticoagulation dosage, bleeding episodes, vaccination status, and death. Patient age was categorized into three groups: less than 40 years, 40–60 years, and greater than 60 years. Then, BMI was categorized into three groups: less than 25, 25–30, and greater than 30.

### 2.2 D-dimer

D-dimer was categorized into two different groups to evaluate its association with patient death: less than 2 µg/mL, greater than or equal to 2 µg/mL versus less than 4 µg/mL, and greater than or equal to 4 µg/mL. The normal D-dimer level was less than 0.50 µg/mL. As a part of the chart review, D-dimer values, corresponding oxygen requirements, and hospitalization day were obtained from each patient’s medical record. D-dimer values were used in two separate data analyses. The highest D-dimer levels and corresponding oxygen requirement (L/min) for each patient were used for the first data analysis in this study. The second set of data analysis included all daily D-dimer levels for each patient, corresponding oxygenation requirements, and associated hospitalization day. Subsequently, hospitalization days were categorized into 11 groups which included days 1–2; days 3–5; days 6–8; days 9–11; days 12–14; days 15–17; days 18–20; days 21–23; days 24–26; days–29; and more than 30 days. This hospitalization category was used to compute the mean and standard error of D-dimer values and oxygenation requirements related to the hospitalization categories. If variable oxygenation requirements were noted for the corresponding day, the highest level was chosen for the analysis.

### 2.3 Bleeding episode

Any bleeding episodes that occurred during hospitalization were noted. This information was obtained from the physician’s progress note or if the patient’s anticoagulation was discontinued.

### 2.4 Vaccination

Vaccination against COVID-19 was first released to the public in the United States in December 2020. A patient is defined as vaccinated if received one or two doses of the COVID-19 vaccine. Only one patient received three doses of the vaccine. Unknown vaccination status was noted for 15 patients where no vaccination status was mentioned in the patient chart. Patients were also reluctant to disclose vaccination status, fearing prejudicial treatment for being unvaccinated.

### 2.5 Anticoagulation

Anticoagulation dosage was categorized into three groups: low dose (Enoxaparin 40 mg SubQ daily or 5,000 units Heparin IV three times a day); moderate dose (Enoxaparin 40 mg SubQ twice a day), or high dose (Enoxaparin 1 mg/kg SubQ twice a day; 25,000 units Heparin IV drip; all doses of direct oral anticoagulation agents). Daily anticoagulation doses were obtained from the physician’s daily progress notes.

### 2.6 Linear regression

The dependent variable used in the linear regression model was oxygen requirements. The independent variables were sex, age, race, BMI, daily D-dimer values, associated oxygenation requirements, modalities of anticoagulation dosage, bleeding episodes, and vaccination status. The oxygenation requirement used in the analysis was related to the maximum D-dimer level for any given patient. The backward selection method was used to find the fitted model.

### 2.7 Logistic regression

The dependent variable used in the logistic regression analysis was patient survival status. The independent variables were sex, age, race, BMI, daily D-dimer values, associated oxygenation requirements, modalities of anticoagulation dosage, bleeding episodes, and vaccination status. All of the variables were entered into the model using the backward elimination method.

Statistical software Stata was used for data analysis.


**Ethical approval:** The research related to human use has been complied with all the relevant national regulations, institutional policies, and in accordance with the tenets of the Helsinki Declaration and has been approved by the authors’ institutional review board of Jackson Hospital, Montgomery, Alabama.
**Informed consent:** All patients were admitted to the hospital as they met hospitalization criteria and gave signed consent to treatments with any FDA-approved drugs.

## 3 Results

The study reviewed 100 COVID-19 patients treated at Jackson Hospital, Alabama (United States), from January 1, 2021, to February 15, 2022.

Table 1 illustrates the demographic characteristics of the 100 patients analyzed in this study. 46% patients were male. For example, among males, 72% survived.

**Table 1:** Demographics characteristics of patient population by survival and death rates

	Variables	Survived	Died
Gender category	*N* = 100	68	22
	Male (*n* = 46)	72% (*n* = 33)	28% (*n* = 13)
	Female (*n* = 54)	87% (*n* = 45)	13% (*n* = 9)
Age category	*N* = 100		
	Age >60 (*n* = 52)	71% (*n* = 37)	29% (*n* = 15)
	Age 40–60 (*n* = 32)	90% (*n* = 29)	10% (*n* = 6)
	Age <40 (*n* = 13)	92% (*n* = 12)	8% (*n* = 1)
BMI category	*N* = 100		
	Low BMI (*n* = 11) (<25)	64% (*n* = 7)	36% (*n* = 4)
	Medium BMI (*n* = 16) (25–30)	75% (*n* = 12)	25% (*n* = 4)
	High BMI (*n* = 53) (>30)	79% (*n* = 42)	21% (*n* = 11)

Table 2 illustrates the clinical parameters of the 100 patients analyzed in this study. D-dimer was evaluated, and different cut-offs were tested to construct the most effective binary variable for whether D-dimer was high and the strength of such a variable as a predictor for death. The binary with a cut-off at 2 µg/mL gave a sharper contrast between low and high D-dimer predictive effects. Among those patients who had less than 2 µg/mL D-dimer, 90% survived; whereas, among those patients with greater than 2 µg/mL D-dimer, 35% died. Among those patients who had a bleeding episode during hospitalization (13 out of 100), 77% of them died. Among those patients who died, 14% received moderate dose anticoagulation; 50% received high-dose anticoagulation, and 36% received low-dose anticoagulation.

**Table 2:** Clinical parameters of patient population by survival and death rates

	Variables	Survived	Died
D-dimer category (Version 1)	*N* = 100	(*n* = 78)	(*n* = 22)
	<2 µg/mL (*n* = 52)	90% (*n* = 47)	10% (*n* = 5)
	≥2 µg/mL (*n* = 48)	65% (*n* = 31)	35% (*n* = 17)
D-dimer category (Version 2)	*N* = 100		
	<4 µg/mL (*n* = 70)	84% (*n* = 59)	16% (*n* = 11)
	≥4 µg/mL (*n* = 30)	63% (*n* = 19)	37% (*n* = 11)
Bleeding status	*N* = 100		
	Bled (*n* = 13)	23% (*n* = 3)	77% (*n* = 10)
	Not bled (*n* = 87)	86% (*n* = 75)	14% (*n* = 12)
Vaccination status	*N* = 100		
	Vaccinated (*n* = 17)	82% (*n* = 14)	18% (*n* = 3)
	Not vaccinated (*n* = 68)	75% (*n* = 51)	25% (*n* = 17)
	Unknown vaccination (*n* = 15)	87% (*n* = 13)	13% (*n* = 2)
Anticoagulation dose	*N* = 100		
	Low dose (*n* = 21)	62% (*n* = 13)	38% (*n* = 8)
	Medium dose (*n* = 47)	94% (*n* = 44)	6% (*n* = 3)
	High dose (*n* = 32)	66% (*n* = 21)	34% (*n* = 11)

Table 3 illustrates the linear regression model demonstrating the predictive effect of vaccination status, D-dimer level, and bleeding status on oxygen requirements among patients with COVID-19, compared to. These three variables were found to be statistically significant with a value of <0.01 and *R* square value of the model was 45%. Among the patients who had a D-dimer value ≥2 µg/mL, oxygen requirements tended to be 31 L/min higher than those with a D-dimer <2 µg/mL (*p* < 0.01). Patients who had bleeding episodes during their hospitalization also were predicted to have a statistically higher (*p* < 0.01) oxygen demand of over 41 L/min compared to those who did not bleed.

**Table 3:** Linear regression demonstrating effect of vaccination, D-dimer, and bleeding episodes on oxygen requirements among patient with COVID-19

Variables	Beta (±SE)
Vaccinated vs Unvaccinated	0.819
	(±7.771)
Uncertain vaccination vs Unvaccinated	21.20***
	(±7.967)
D-dimer ≥2 vs <2	31.65***
	(±5.885)
Bled vs not bled	41.11***
	(±8.513)
Constant	7.546
	(±4.618)
Observations	100
*R*-squared	0.445

Standard errors in parentheses. Fixed effects model omits unvaccinated participants.

***p < 0.01.

Table 4 illustrates the logistic regression model demonstrating the predictive effect of D-dimer level and bleeding status on COVID-19 patient survival. This finding indicates that patients with a D-dimer level <2 are 76% less likely to die compared to patients with maximum D-dimer of ≥2. The study also shows that those patients who did not bleed were 94% less likely to die from COVID-19 compared to those who bled due to a high dose of anticoagulation. The linear regression model also found similar findings, but in the logistic regression model, vaccination status was not found to be significant.

**Table 4:** Logistic regression demonstrating effect of D-dimer, and bleeding episodes on patient survival

Variables	Survival odds ratio
D-dimer ≥2 vs <2	0.24**
	(±0.15)
Bled vs not bled	0.06***
	(±0.04)
Constant	13.68***
	(±7.25)
Observations	100

Standard errors in parentheses.

***p< 0.01, **p < 0.05.

Table 5 illustrates the analysis of the mean D-dimer and corresponding oxygen requirements where D-dimer levels were categorized into 11 hospital days categories (ranks). The D-dimer levels were noted to follow a similar trend in which the values peaked at the maximum point of illness at around day 24 to day 26 and decreased subsequently. The associated oxygen requirements follow the same trend like the D-dimer values during hospitalization. The lowest mean oxygen requirement was during the hospital days 1 and 2 at a value of 18.6 L/min with a corresponding D-dimer mean value of 2.7 μg/mL. The peak mean oxygen requirement was during the hospital days 21 and 23 at a value of 78.9 L/min with a corresponding D-dimer mean value of 5.8 μg/mL. The peak D-dimer level was during hospital days 24–26 at a value of 7.0 μg/mL with a corresponding oxygen requirement of 77.1 L/min.

**Table 5:** Mean D-dimer and corresponding oxygen requirement are shown by hospitalization days category among patients with COVID-19

Hospitalization day categories	Hospital days	Sample size (*n*)	D-dimer (μg/mL)	Oxygen requirement (L/min)
			Mean (±SE)	Mean (±SE)
1	1–2	135	2.7 (±0.4)	18.6 (±2.4)
2	3–5	135	2.1 (±0.3)	25.1 (±2.6)
3	6–8	101	2.6 (±0.4)	36.1 (±3.3)
4	9–11	77	3.1 (±0.4)	48.1 (±3.7)
5	12–14	64	3.7 (±0.6)	47.8 (±4.0)
6	15–17	54	3.6 (±0.7)	46.1 (±5.2)
7	18–20	30	4.3 (±0.8)	55.4 (±7.8)
8	21–23	16	5.8 (±1.6)	78.9 (±9.0)
9	24–26	12	7.0 (±2.3)	77.1 (±8.9)
10	27–29	9	2.2 (0.4)	62.0 (15.1)
11	>30 days	14	3.3 (0.5)	29.5 (12.4)

**Figure 1:**
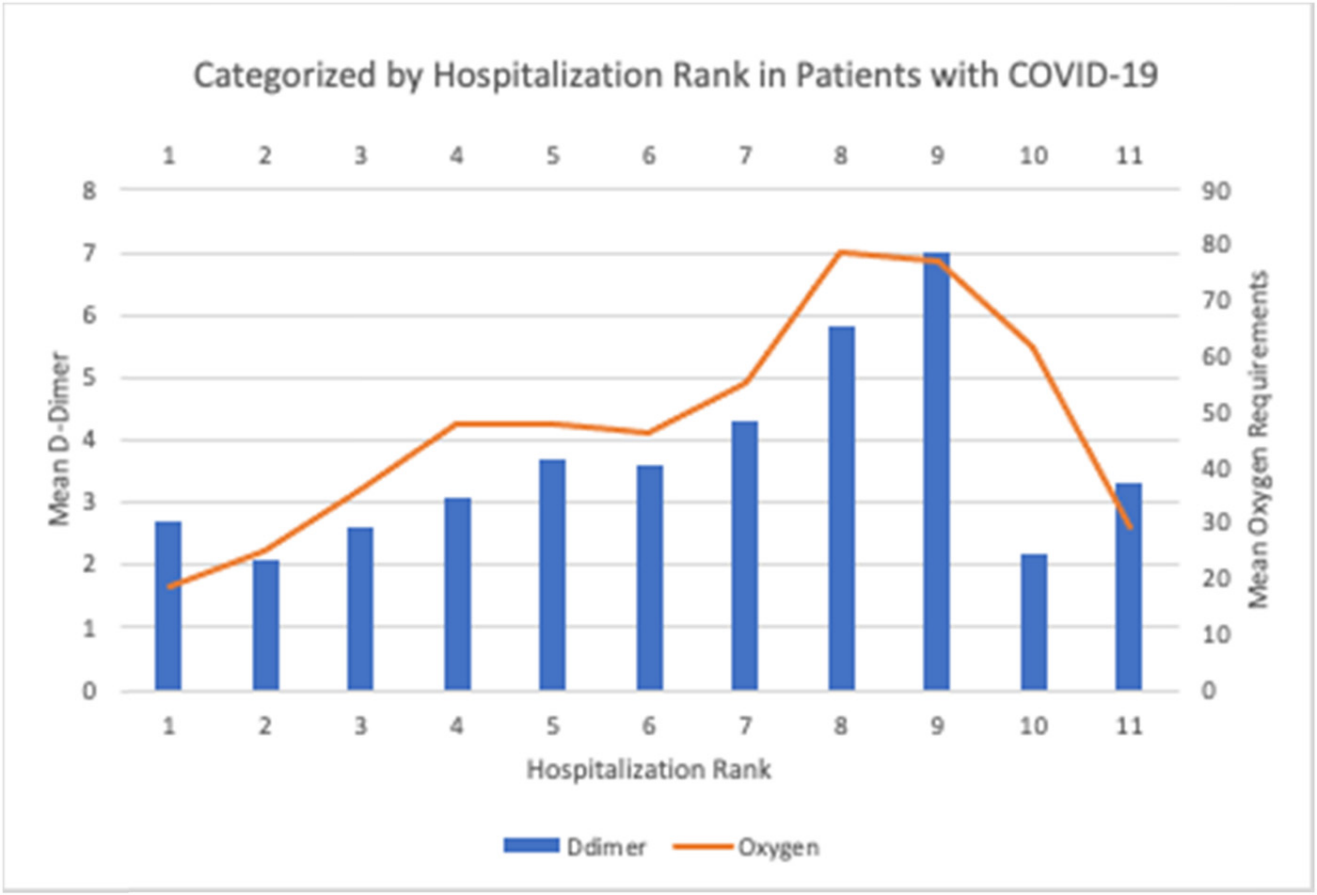
Mean D-dimer and mean oxygen requirements categorized by hospitalization rank in patients with COVID-19.

Figure 1 illustrates the trend of rising D-dimer values corresponding to hospitalization days and oxygenation demand until a peak is reached where the D-dimer value rapidly decreases along with the oxygenation demand.

This association is tested using polynomial fit regression. The results depicted in this Figure 1 indicate that patients diagnosed with COVID-19 exhibit lower levels of D-dimer at the time of diagnosis or early stages of hospitalization. However, as the disease progresses, the D dimer levels gradually increase. Concurrently, the associated oxygen requirements also show a continuous upward trend, mirroring the patterns of D-dimer levels, reaching their peak around days 24–26. Following this peak, the D-dimer levels start to decline, which is also accompanied by a reduction in oxygen requirements. These findings suggest a correlation between D-dimer levels and the severity of the disease, highlighting its potential as a biomarker to monitor disease progression and response to treatment.

## 4 Discussion

Whereas the three randomized clinical trials, ATTACC, ACTIV-4a, and REMAP-CAP only checked a baseline D-dimer, this study evaluated only those patients who had multiple D-dimer tests during the course of hospitalization. The study suggests that daily D-dimer trend is the direct indicator of patients’ prognosis in patients with COVID-19. This study also found that a D-dimer level ≥2 µg/mL is strongly associated with increased death in the multivariate logistic regression model (*p*-value <0.05) and increased oxygenation requirements in the multivariate linear regression model (*p*-value <0.01). The study found that 77% of the patients who had a D-dimer ≥2 µg/mL, died during hospitalization. In this study, the oxygen requirements and D-dimer trends were found following similar trends and directions both in univariate and multivariate linear analyses. In a previous study, this trend held consistent at the individual level [5].

The D-dimer can be elevated or abnormal at baseline for many patients due to pre-existing chronic medical conditions. This is likely why D-dimer trends, instead of depending on baseline D-dimer (i.e. changes of D-dimer as opposed to day one level), are more meaningful for patients prognosis. Dr. Kabir previously reported that D-dimer values continue to increase prior to patient death among patients with COVID-19 [5]. This can be mediated by increasing D-dimer which indicates increased thrombogenic activity due to COVID-19. The current study also showed that decreasing trends of D-dimer, when treated with appropriate dose of anticoagulation, are associated with decreased oxygen requirements which lead to patient survival. While patients are having increasing trends of D-dimer, keeping the patient with the same dose of anticoagulation would lead to increase in D-dimer and oxygen requirements and subsequently death. Hence, a baseline D-dimer should be established at admission and then daily D-dimer trends should guide anticoagulation dose to reduce death related to COVID-19 instead of treating everyone with therapeutic anticoagulation.

Dr. Kabir’s previously reported that non-critically ill COVID-19 patients who were already on direct oral anticoagulants prior to the diagnosis of COVID-19 had increased survival rates [6]. This current study has found that non-critically ill patients with COVID-19 who were treated with intermediate-dose anticoagulation at an early stage have a higher survival rate compared to those who received low- or high-dose anticoagulation. The study also found that patient’s D-dimer levels were significantly associated with patient oxygen requirements and patient survival. Since patients treated with low- or high-dose anticoagulation were associated with increased death, the treatment strategies should be to start the patient on moderate-dose anticoagulation and gradually titrate the dose up or down based on the patient’s daily D-dimer trends and bleeding risk. Dr. Kabir’s treatment strategy for COVID-19 was also published in 2021 [5].

The current study found that the best treatment strategy in non-critically ill COVID-19 patients was to start moderate dose anticoagulation (Enoxaparin 40 mg SQ twice a day) and titrate the dose up or down based on D-dimer trends and patient’s condition. The death rate among patients treated with moderate dose D-dimer was six percent.

Treating patients with anticoagulation is only beneficial when capillaries are still open, not clogged due to microthrombi. Therefore, the patients who present in the hospital with nasal cannula oxygen, high flow nasal cannula oxygen, or patients with moderately high D-dimer (less than two-fold higher than the upper limit of normal) usually survive with anticoagulation and steroid combination treatments. The authors observed that increasing trends of D-dimer were inversely related to the survival rates. There is a dose–effect relationship between the decreasing trends of D-dimer with the anticoagulation dose. Hence, D-dimer trends can be considered to predict patient prognosis. This finding has also been correlated by another study stating that D-dimer monitoring will be a crucial approach in the clinical practice of COVID-19 infection [7]. It is also possible that when the widespread coagulation cascade is initiated at the onset of COVID-19 inoculation in patients with COVID-19 who are critically ill, they do not respond to therapeutic anticoagulation. The suggested reason behind the lower chance of recovery is that anticoagulation does not dissolve microthrombi (blood clots) but rather prevents their further growth.

This research delves into the fundamental relationship between COVID-19-related deaths and widespread multiorgan thromboembolic activity. We propose that the primary mechanism of death in these cases is the occlusion of the microvascular bed by thromboembolism, leading to hypoxia and subsequent organ failure.

In contrary to the popular belief that underlying conditions like COPD, diabetes mellitus, coronary artery disease, congestive heart failure, or a history of cerebrovascular accident may influence thromboembolic activity, we argue that it is hypercoagulable states, such as atrial fibrillation, a history of deep vein thrombosis, and PE, that are more directly related to accelerated thromboembolic activity. Given the thrombotic nature of death, the study did not include the traditional cardiovascular risk factors to predict COVID-19 mortality. Instead, our focus was on understanding the role of thrombosis in mortality outcomes.

In Table 2, we clearly demonstrate that patients with lower D-dimer levels have significantly higher survival rates compared to those with higher D-dimer levels. This finding suggests that D-dimer levels can serve as a prognostic indicator for COVID-19 patients.

Furthermore, in Table 5, we present data on the mean D-dimer levels across different hospital days. We observe a consistent increase in mean D-dimer levels from hospital day 1 to hospital day 25. This trend indicates that as COVID-19 progresses and formulates thromboembolism in the microvascular bed, D-dimer levels tend to rise over time. This observation supports the notion that a lower D-dimer level at the time of diagnosis may be an indicator of an earlier stage of COVID-19 presentation. A previous study also demonstrated that patients presenting with oxygen desaturation upon admission exhibited notably elevated D-dimer levels compared to those with normal oxygen saturations [8]. This suggests that lower D-dimer levels may be associated with the early stages of the disease.

Based on these findings, we propose that earlier diagnosis with the capture of D-dimer levels can serve as a good predictor for patient survival. Detecting COVID-19 at an earlier stage allows for the timely initiation of appropriate interventions, including anticoagulation therapy. We speculate that initiating anticoagulation earlier in the disease course, particularly in patients with higher D-dimer levels, may contribute to higher survival rates by reducing the impact of excessive thromboembolic activity.

It is important to note that while our analysis supports the potential benefits of early diagnosis and anticoagulation, further research is warranted to establish the optimal timing and dosage of anticoagulant therapy in COVID-19 patients. Additionally, the interplay between D-dimer levels, disease severity, and the efficacy of anticoagulation warrants further investigation.

In addition, the previous study also showed the rate for major thrombotic events or death among non-critical COVID-19 patients admitted to hospital medical floors was 9.9% (104 patients out of 1,046) compared to those admitted to intensive care units of 41.1% (230 patients out of 560), this indicates that initiation of anticoagulation in an earlier stage, even if it is lower dose, leads to better survival, most likely due to these patients’ admission to the hospital floor at an earlier stage of the disease process. The survival rate among non-critically ill patients on usual care thromboprophylaxis is higher at 91.8% (962 patients out of 1,048) compared to 64.5% (364 patients out of 564) in critically ill patients receiving the same treatment [1,2].

Vaccination has improved the outlook for survival in COVID-19 patients, and higher rates of vaccination have been shown to be associated with reduced death rates. A study on the impact of the Pfizer-BioNTech COVID-19 vaccine showed a 51% reduction in death in individuals who had received one dose [9]. The vaccine works by inducing a strong S protein antibody response and a CD4+ T cell response. This thereby reduces the viral load and associated vasculitis causing a decrease in coagulopathy [10].

### 4.1 Limitations

One of the most significant limitations of the above study is its retrospective design. As a retrospective study, it relies on existing data collected for other purposes, and as such, it may be subject to various biases and confounding factors that could affect the results.

To address this limitation and further validate the study findings, we highly recommend considering a prospective clinical trial. Conducting a clinical trial would allow for a more controlled and systematic approach to data collection, ensuring standardized protocols and minimizing potential biases. Additionally, a clinical trial could offer an opportunity to explore causality and establish a more robust cause-and-effect relationship between COVID-19, thromboembolic activity, and mortality outcomes. By conducting a clinical trial, we can enhance the strength of the evidence supporting our findings and increase the generalizability and applicability of the study results in a clinical setting.

## 5 Conclusion

The study concludes daily D-dimer trends can predict COVID-19 patient survival or daily oxygen requirements. This indicates that D-dimer can be considered the miracle molecule for COVID-19 patients’ prognosis. In addition, a moderate dose of anticoagulation is superior to high-dose or low-dose anticoagulation if it is started at an early stage of COVID-19 diagnosis. The study recommends starting anticoagulation at a moderate dose and titrating the anticoagulation dose based on D-dimer trends to reduce the risk of life-threatening bleeding. The study recommends developing bleeding risk-based COVID-19 anticoagulation treatment strategies so that patients with high bleeding risk can be spotted initially and considered a lower dose of anticoagulation. Further clinical trials are recommended to evaluate patients’ outcome while treated with moderate dose or low dose direct oral anticoagulation (e.g. Apixaban or Rivaroxaban) at the diagnosis of COVID-19 among patients who do not require any oxygen.
